# Computational Analysis of Periplasmic Protein-Mediated Resistance to Membrane Extraction of a Trimeric Autotransporter Adhesin Transmembrane Domain

**DOI:** 10.34133/csbj.0045

**Published:** 2026-04-08

**Authors:** Jun Sasahara, Shogo Yoshimoto, Atsuo Suzuki, Katsutoshi Hori

**Affiliations:** Department of Biomolecular Engineering, Graduate School of Engineering, Nagoya University, Nagoya, Aichi 464-8603, Japan.

## Abstract

Gram-negative bacteria display large surface proteins exposed to substantial mechanical stresses during surface attachment, fluid flow, and physical perturbations. AtaA, a trimeric autotransporter adhesin from *Acinetobacter* sp. Tol 5, forms long homotrimeric fibers (~260 nm) that mediate robust adhesion. Its C-terminal transmembrane (TM) domain anchors the fiber to the outer membrane and also associates with the N-terminal domain of the periplasmic auxiliary protein TpgA (TpgA-N). However, the mechanical importance of this interaction remains elusive. Here, we quantified TpgA-mediated stabilization of AtaA-TM anchoring using all-atom steered molecular dynamics and free energy calculations in an asymmetric *Acinetobacter* outer membrane model. TpgA-N binding significantly enhanced resistance to membrane extraction, approximately doubling the peak force required compared with AtaA-TM alone. Structural analyses revealed that multiple electrostatic contacts at the AtaA-TM–TpgA-N interface collectively reinforce the complex under tensile loading. Furthermore, thermodynamic integration yielded a favorable restrained separation free energy, supporting stabilization of the complex. Because TpgA-N is a hydrophilic periplasmic protein, its translocation through the hydrophobic membrane core is energetically unfavorable. Thus, membrane extraction of AtaA-TM necessitates the prior dissociation of the stable AtaA-TM–TpgA-N interaction. Together, our results demonstrate that TpgA mechanically reinforces the membrane anchor of AtaA through both thermodynamic stabilization and increased resistance to tensile forces. This study highlights a specialized strategy where a periplasmic protein strengthens the membrane anchoring of giant bacterial adhesins to withstand environmental loads.

## Graphical Abstract

**Figure:**
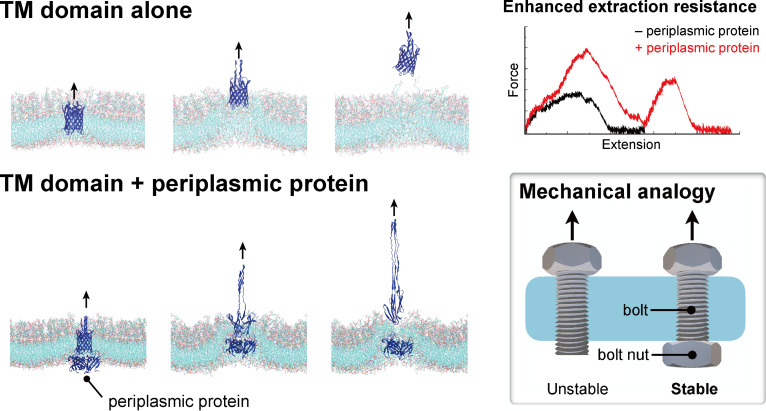

## Introduction

Gram-negative bacteria possess a multilayered cell envelope composed of an inner membrane, a peptidoglycan layer, and an outer membrane [1,2]. Although several outer membrane proteins are known to interact noncovalently with the peptidoglycan layer, the outer membrane as a whole is not covalently integrated into the peptidoglycan network and exhibits distinct mechanical properties associated with its asymmetric organization [2,3]. Despite this lack of direct structural support, Gram-negative bacteria frequently display large surface proteins that experience mechanical stress arising from fluid flow, surface attachment, and physical perturbations [4–6].

Outer membrane proteins, particularly surface-exposed adhesins, are not only involved in molecular recognition or transport but also required to withstand mechanical load [4,7]. These surface-associated assemblies are frequently subjected to long-range pulling forces [5,8], and their ability to withstand such forces is closely linked to their biological function [7,9]. Understanding how mechanical forces are transmitted through the outer membrane and resisted at the molecular level remains a fundamental challenge in bacterial structural biology.

Trimeric autotransporter adhesins (TAAs) represent a major class of large, surface-exposed outer membrane adhesins in Gram-negative bacteria [10–12]. They are widely distributed and form elongated homotrimeric fibers that can extend tens to hundreds of nanometers from the cell surface [13–15]. Typically, TAAs are composed of a globular head domain and a long stalk containing various conserved β-structured motifs interleaved with coiled coils; the complex is anchored to the outer membrane by a conserved C-terminal β-barrel transmembrane (TM) domain. During adhesion under shear flow or surface detachment, these extended fibers are expected to experience substantial tensile forces [5,8]. Previous studies have shown that the extracellular domains of TAAs, including head and stalk regions, possess structural features that contribute to mechanical robustness [15–17]. For example, work on YadA has demonstrated that trimeric assembly and interchain interactions play key roles in distributing applied force along the fiber [16,17].

In contrast to the growing understanding of extracellular TAA domains, the mechanical behavior of membrane anchoring regions has remained largely unexplored. From a structural perspective, the TM domain represents the final point at which forces transmitted through the extracellular fiber are applied to the outer membrane. Recent molecular dynamics (MD) studies have substantially advanced our understanding of the structure and dynamics of the outer membrane, including outer membrane proteins embedded in an asymmetric lipid environment whose outer leaflet is composed of lipopolysaccharides (LPSs) [18]. However, how such membrane-embedded anchoring regions respond to tensile forces remains unclear. Because the outer membrane is fluid and deformable, how tensile forces are accommodated at the TM interface has not been established. Regardless of how mechanical stress is distributed along the passenger domain, the overall stability of a large adhesin ultimately depends on how these forces are accommodated at the membrane interface.

AtaA from *Acinetobacter* sp. Tol 5 is a TAA that mediates strong, nonspecific adhesion to various material surfaces and forms fibers approximately 260 nm in length [19,20]. Recent structural studies have revealed that the AtaA TM domain forms a stable complex with a peptidoglycan-binding periplasmic auxiliary protein, TpgA [21]. This 2-component anchoring arrangement, combined with the fiber’s length of over 200 nm, makes the AtaA–TpgA complex an ideal model for studying the anchoring mechanics of TM domains under tensile load. In this study, we provide mechanistic insights into the TpgA-mediated stabilization of AtaA-TM anchoring by performing all-atom steered molecular dynamics (SMD) simulations [22] and free energy calculations in an asymmetric outer membrane model.

## Materials and Methods

### Modeling of simulation systems

Simulation systems were constructed with CHARMM-GUI [23] based on the crystal structure of the trimeric AtaA transmembrane (AtaA-TM) domain in complex with its periplasmic peptidoglycan-binding protein TpgA-N [Protein Data Bank (PDB) ID: 9VNJ]. To establish simulation conditions, an initial AtaA-TM–TpgA-N model containing the complete helical stalk fragment present in the crystal structure of the AtaA-TM–TpgA-N complex was prepared. Subsequently, a variant with a truncated helical stalk was generated by removing residues 3,523 to 3,556 from the N-terminal region of the stalk; this construct is referred to as short-stalk AtaA-TM. Using the short-stalk AtaA-TM model, 2 simulation systems were prepared: (a) a short-stalk AtaA-TM–TpgA-N complex and (b) a short-stalk AtaA-TM system lacking TpgA-N.

In all simulation systems, the AtaA-TM was embedded in an asymmetric lipid bilayer. The outer leaflet consisted of LPS derived from *Acinetobacter baumannii*, modeled as a smooth LPS chemotype with the following glycan sequence: α-D-Glc (1→2) β-D-Glc (1→4) β-D-Glc (1→4) β-D-Glc (1→3) [β-D-GalN (1→4)] α-D-GlcNAc (1→4) [β-D-GlcN (1→7)] α-D-Kdo (2→5) [α-D-Kdo (2→4)] α-D-Kdo (2→lipid A). The LPS model included O-antigen chains represented by a truncated number of repeating units. Calcium ions (Ca^2+^) were added to neutralize the negative charges of lipid A and the core oligosaccharide. The inner leaflet consisted of a 4:1 mixture of 1-palmitoyl-2-oleoyl-*sn*-glycero-3-phosphoethanolamine (POPE) and 1-palmitoyl-2-oleoyl-*sn*-glycero-3-phosphatidylglycerol (POPG). The CHARMM36m force field [24] in combination with the CHARMM-modified TIP3P water model [25] was used to describe all systems. Sodium chloride was added to a final concentration of 0.15 M. Details of the computational systems are summarized in Table S1. For free energy calculations described in the Restrained separation free energy calculation section, a simulation system of the AtaA-TM–TpgA-N complex was constructed using the same structural models and membrane compositions, with the primary difference being the size of the simulation box (Table S2).

### MD simulations

All MD simulations were performed using GROMACS 2020 [26]. The temperature was maintained at 301.15 K using the V-rescale thermostat [27], and the pressure was controlled at 1 bar using the Parrinello–Rahman barostat [28]. All bonds involving hydrogen atoms were constrained using the LINCS algorithm [29]. Long-range electrostatic interactions were calculated using the particle mesh Ewald (PME) method [30], with a real-space cutoff distance of 1.2 nm.

Prior to SMD simulations, all systems were equilibrated using a multistep protocol following the standard CHARMM-GUI membrane equilibration procedure. This protocol consisted of energy minimization followed by 6 sequential equilibration stages in which positional restraints on proteins and lipids were gradually released. The equilibration workflow is summarized in Table S3, and the GROMACS parameter files (.mdp) used in each step are provided in the Supplementary Materials (Texts S1 to S7).

The final equilibrated structure served as the starting configuration for SMD simulations. Individual SMD simulations were initiated from the same equilibrated structure, with randomized initial velocities assigned using different random seeds to generate statistically independent trajectories. The specific random seed values used for each SMD simulation are listed in Table S4. SMD simulations were conducted by defining the reaction coordinate as the distance between the center of mass (COM) of the N-terminal atoms of the AtaA-TM domain and the COM of the lipid membrane. Pulling was performed along the *Z* axis, perpendicular to the membrane plane, using a harmonic potential at a constant velocity of 2 nm ns^−1^ or 1 nm ns^−1^ with a spring constant of 1,000 kJ mol^−1^ nm^−2^. The force acting on the pulling coordinate was recorded throughout the simulation, and force–extension profiles were obtained from the dependence of force on the pulling distance. Additional SMD simulations were performed with the trimeric TpgA-N alone using the same membrane composition and pulling protocol as used in the AtaA-TM–TpgA-N simulations. Details of this system are summarized in Table S1. Interfacial contacts among AtaA-TM, TpgA-N, and the membrane were quantified during membrane extraction. Contacts were defined as atom pairs between the respective components separated by 0.45 nm or less, with hydrogen atoms included in the calculation. All trajectories were visualized using VMD [31].

### Restrained separation free energy calculation

The free energy calculation was designed to quantify the AtaA-TM–TpgA-N interaction by defining the separation between the TpgA-N domain and the membrane-embedded AtaA-TM domain along the membrane normal as the reaction coordinate. The restrained separation free energy between AtaA-TM and TpgA-N was evaluated by systematically varying the COM distance between the 2 domains along the membrane normal. To generate the sampling windows, the TpgA-N domain was translated along the *Z* axis in increments of 0.05 nm relative to AtaA-TM, yielding 23 sampling windows. For each window, a 30-ns MD simulation was performed. For each COM separation, positional restraints were applied to maintain the target AtaA-TM–TpgA-N COM distance. In addition, rotational motions in the *XY* plane were restrained using the rm2-pf rotational restraint potential to prevent lateral reorientation of the complex while allowing separation along the *Z* axis [32]. The mean force acting along the COM separation direction (*Z* axis) was calculated for each separation window by averaging over the equilibrated portion of the trajectory (2 to 30 ns). To assess statistical convergence and estimate uncertainty, block averaging was performed within each window. The 2- to 30-ns trajectory was divided into 7 consecutive blocks of 4 ns each, and the mean force was calculated for each block. Thermodynamic integration [33,34] was then performed separately for each block to obtain block-wise free energy profiles along the COM separation coordinate. The 95% confidence interval (CI) at each separation distance was estimated using a nonparametric percentile method, defined by the 2.5th and 97.5th percentiles of the block-wise free energy profiles. The cumulative free energy profile was obtained by thermodynamic integration of the window-averaged mean forces over the COM distance. Because positional and orientational restraints were applied during the simulations, the resulting free energy corresponds to a restrained separation free energy along the chosen reaction coordinate rather than a standard-state binding free energy.

## Results and Discussion

### SMD simulation setup and extraction behavior of AtaA-TM

**Fig. 1.**
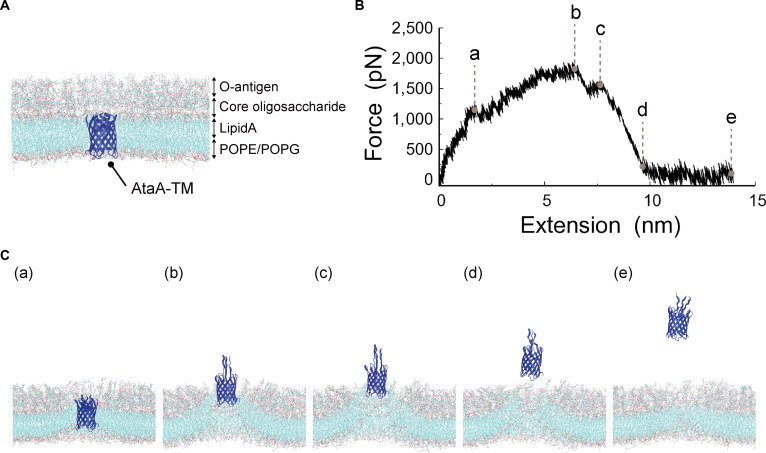
SMD simulation of the short-stalk AtaA-TM during membrane extraction. (A) Initial configuration of the AtaA-TM model, in which the coiled-coil region of the AtaA-TM domain was truncated and embedded in a lipid bilayer. Water molecules and ions are omitted for clarity. (B) Force–extension profile obtained from the SMD simulation in which the AtaA-TM model was extracted along the membrane normal (*Z* axis). Points a to e indicate representative positions along the force–extension profile. (C) Structural snapshots of the AtaA-TM model corresponding to these points.

We first examined the mechanical response of the trimeric AtaA-TM–TpgA-N complex, which contains the complete helical stalk fragment resolved in the crystal structure of the AtaA-TM–TpgA-N complex (PDB ID: 9VNJ), using SMD simulations. The asymmetric lipid bilayer was constructed using LPS from *A. baumannii* in the outer leaflet and a POPE/POPG mixture in the inner leaflet, based on reported lipid compositions of *Acinetobacter* species [35] (Fig. S1A). Upon application of tensile loading normal to the membrane, unfolding of the extended helical stalk region on the extracellular side was observed prior to any detectable extraction of the membrane-embedded AtaA-TM β-barrel (Fig. S1B and C and Movie S1). During this process, the tensile deformation predominantly occurred as unfolding of the helical stalk, and extraction of the membrane-embedded AtaA-TM β-barrel from the lipid bilayer was not observed within the simulated time scale. These observations indicate that, in the AtaA-TM–TpgA-N system, the long helical stalk dominates the mechanical response under tensile load. Structural inspection of the trajectories further indicates that extension is primarily accommodated by progressive elongation and partial unfolding of the coiled-coil stalk, while the membrane-embedded β-barrel remains structurally intact with minimal deformation throughout the simulation. Consequently, the force required for membrane extraction of the AtaA-TM domain itself cannot be directly evaluated in this configuration. To reduce computational cost and to enable efficient sampling of AtaA-TM extraction events, subsequent SMD simulations were therefore performed using a short-stalk AtaA-TM model, in which the N-terminal helical stalk region was truncated (Fig. 1A).

Using the short-stalk AtaA-TM system lacking TpgA-N, we next examined the extraction behavior of the AtaA-TM domain from the membrane (Fig. 1 and Movie S2). In this simulation, the AtaA-TM β-barrel was extracted from the lipid bilayer while largely maintaining its overall structure, accompanied by residual elongation of the remaining helical stalk segment and local deformation of the surrounding membrane. Extraction of the AtaA-TM domain from the membrane occurred after this residual stalk extension was completed, in agreement with the sequence of events observed in simulations using the longer stalk construct. The force–extension profile showed an increase in force with pulling distance, reaching a maximum immediately prior to complete extraction of the AtaA-TM β-barrel from the membrane. In this profile, the peak force reflects the resistance of the membrane-embedded AtaA-TM domain against tensile extraction. Across 5 independent simulations, the extraction behavior was highly reproducible (Fig. S2), yielding a peak extraction force of 1,981 ± 112 pN (mean ± SD, *n* = 5; 95% CI, 1,842 to 2,120 pN). These results demonstrate that the AtaA-TM domain alone possesses substantial mechanical resistance within the membrane while maintaining the intrinsic sequence of mechanical responses observed in the corresponding full-stalk simulations.

### AtaA-TM extraction behavior and enhanced mechanical resistance upon TpgA-N binding

**Fig. 2.**
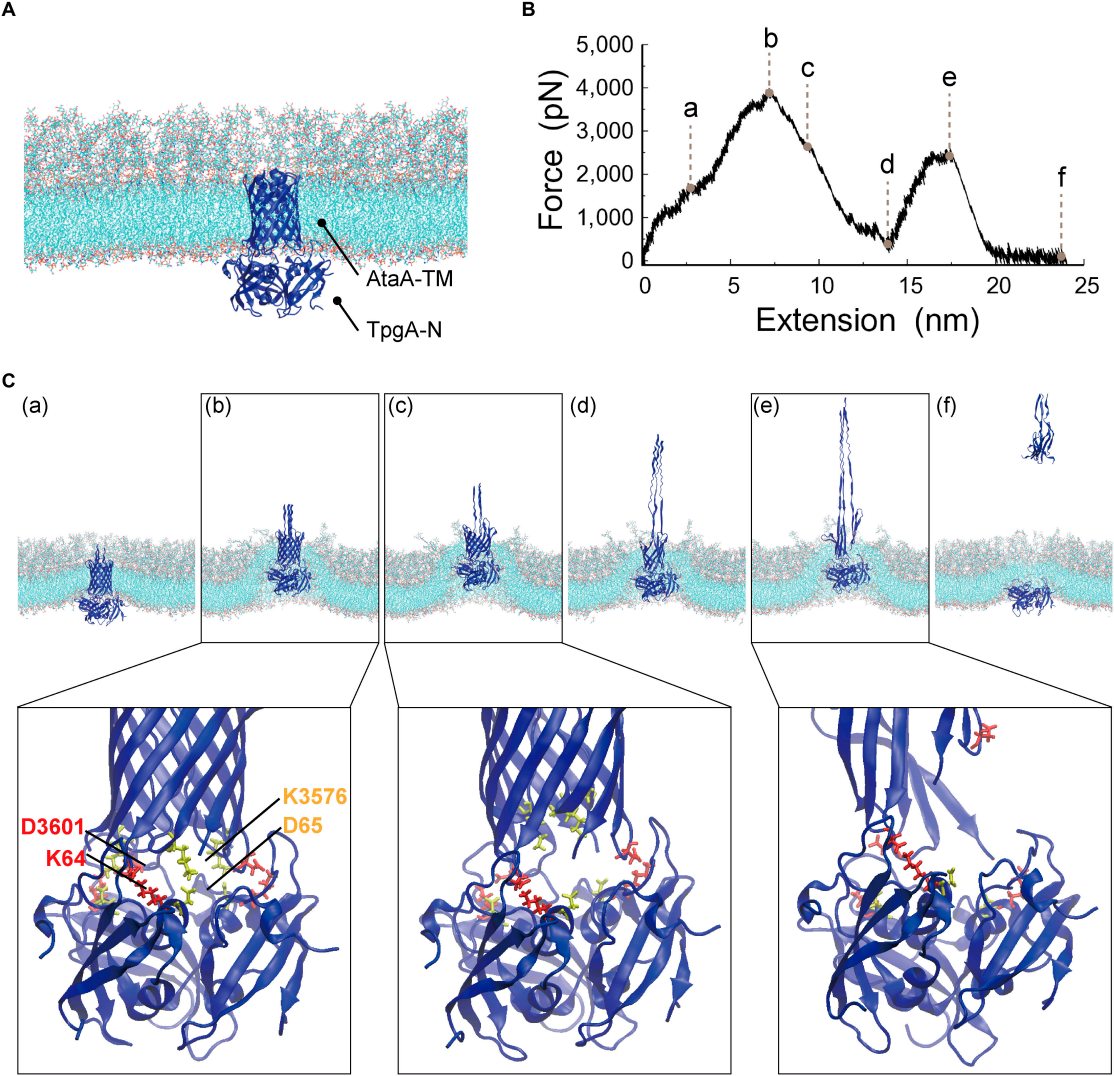
SMD simulation of the short-stalk AtaA-TM–TpgA-N complex during membrane extraction. (A) Initial configuration of the AtaA-TM–TpgA-N complex embedded in a lipid bilayer. Water molecules and ions are omitted for clarity. (B) Force–extension profile obtained from the SMD simulation in which the AtaA-TM–TpgA-N complex was extracted along the membrane normal (*Z* axis). Points a to f indicate representative positions along the force–extension profile. (C) Structural snapshots and enlarged views of the AtaA-TM–TpgA-N complex corresponding to these points. The AtaA-TM loop spanning residues 3,601 to 3,604 and residue K64 of TpgA-N are shown in red, with D3601 and K64 displayed in stick representation. The AtaA-TM loop spanning residues 3,573 to 3,576 and residue D65 of TpgA-N are shown in yellow, with K3576 and D65 displayed in stick representation.

We next examined the membrane extraction behavior of AtaA-TM in the presence of its periplasmic binding partner TpgA-N using the short-stalk AtaA-TM–TpgA-N complex (Fig. 2 and Movie S3). The AtaA-TM–TpgA-N complex exhibited a markedly higher resistance to membrane extraction than the AtaA-TM system. Across 5 independent simulations, the peak force required for the extraction of AtaA-TM in the presence of TpgA-N was 3,855 ± 76 pN (mean ± SD, *n* = 5; 95% CI, 3,761 to 3,949 pN; Fig. S3), which is approximately 2-fold higher than the peak force observed for the AtaA-TM system lacking TpgA-N. This increase demonstrates that complex formation with TpgA-N substantially enhances the mechanical resistance of AtaA-TM against tensile extraction from the membrane.

In our SMD setup, the pulling velocity of 2 nm ns^−1^ combined with a spring constant of 1,000 kJ mol^−1^ nm^−2^ corresponds to a loading rate of approximately 3.3 × 10^12^ pN s^−1^. When the pulling velocity was reduced to 1 nm ns^−1^, the loading rate decreased to ~1.6 × 10^12^ pN s^−1^ (Fig. S4 and Movie S4). These loading rates are several orders of magnitude higher than those typically employed in atomic force microscopy (AFM)-based force spectroscopy (~10^2^ to 10^5^ pN s^−1^) [36,37]. Such high rates are necessary to observe complete extraction of AtaA-TM within computationally accessible timeframes, leading to elevated rupture forces dominated by non-equilibrium effects, such as viscous drag and kinetic barriers. While these absolute magnitudes should not be interpreted as quasi-equilibrium values, an overestimation of rupture forces compared to equilibrium conditions due to non-equilibrium effects is expected, as simulations and experiments probe force-induced processes at different temporal scales. However, our parameters are well-aligned with recent studies of membrane proteins [38,39]; for instance, Jayawardena et al. [39] employed a comparable loading rate of ~4 × 10^12^ pN s^−1^. The qualitative consistency across different velocities confirms that the simulation effectively captures the essential mechanical characteristics of the system, rendering the results suitable for comparative analysis of the stabilizing role of TpgA-N.

Furthermore, the force–extension profiles revealed qualitative differences between the 2 systems. While extraction of AtaA-TM in the absence of TpgA-N was characterized by a single dominant force peak (Fig. 1B), simulations of the AtaA-TM–TpgA-N complex consistently exhibited 2 distinct force peaks during the extraction process (Fig. 2B). These features indicate that membrane extraction proceeds through multiple mechanically distinct steps in the presence of TpgA-N. In the AtaA-TM system lacking TpgA-N, the β-barrel structure of AtaA-TM remained largely intact as it was extracted from the membrane (Fig. 1C). In contrast, in the AtaA-TM–TpgA-N complex, partial deformation of the AtaA-TM β-barrel was observed prior to dissociation from TpgA-N, followed by membrane extraction (Fig. 2C). To evaluate whether TpgA-N itself could be forced into the membrane, additional SMD simulations were performed in which the trimeric TpgA-N structure alone was pulled through the membrane. The peak force required for membrane penetration was 4,352 ± 40 pN (mean ± SD, *n* = 3; 95% CI, 4,252 to 4,452 pN; Fig. S5 and Movie S5), exceeding that required for extraction of the AtaA-TM–TpgA-N complex (3,855 ± 76 pN). These results indicate that insertion of TpgA-N into the membrane is energetically highly unfavorable.

Structural inspection of the AtaA-TM–TpgA-N complex further suggests that the 2 force peaks observed in the force–extension profiles correspond to sequential rupture of 2 interfacial salt bridges: first, between K3576 of AtaA-TM and D65 of TpgA-N, followed by dissociation of the D3601–K64 interaction (Fig. 2C). Time-resolved plots of salt-bridge contacts (K3576–D65 and D3601–K64) as a function of extension showed that in both cases, salt-bridge rupture events align closely with the corresponding force maximum (Fig. S6). These residue pairs are located in the loop regions of AtaA-TM (residues 3,573 to 3,576 and 3,601 to 3,604) and the complementary interface of TpgA-N, and were previously identified as major stabilizing contacts in the crystal structure of the AtaA-TM–TpgA-N complex [21]. The presence of multiple electrostatic contact points at the AtaA-TM–TpgA-N interface suggests that these interfacial interactions cooperatively enhance the mechanical resistance of the complex and that a greater force is required for membrane extraction in the presence of TpgA-N.

### Interfacial interactions and thermodynamic stability of the AtaA-TM–TpgA-N complex

**Fig. 3.**
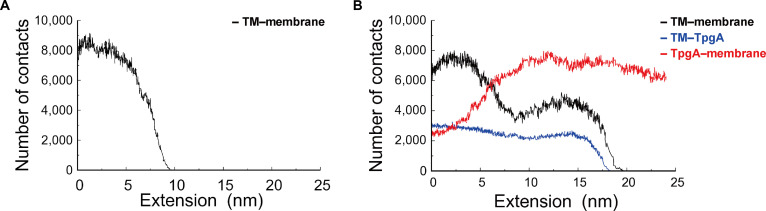
Changes in interfacial contact numbers during AtaA-TM extraction. (A) Number of contacts between the AtaA-TM and the membrane in the AtaA-TM system lacking TpgA-N as a function of extension. (B) Number of contacts between the AtaA-TM and the membrane (black), the AtaA-TM and TpgA-N (blue), and TpgA-N and the membrane (red) in the AtaA-TM–TpgA-N complex system. Contacts were defined as atomic contact pairs within 0.45 nm between the indicated molecular components.

Because TpgA-N is a soluble, hydrophilic periplasmic protein, its direct translocation through the hydrophobic membrane core would require a prohibitively large energetic cost. Consequently, membrane extraction of AtaA-TM in the complex necessarily requires prior dissociation of the AtaA-TM–TpgA-N interaction. We analyzed changes in the number of interfacial contacts among AtaA-TM, TpgA-N, and the membrane during the extraction process (Fig. 3). In the AtaA-TM system lacking TpgA-N, the number of contacts between AtaA-TM and the membrane sharply decreased as extraction proceeded, ultimately resulting in detachment of the AtaA-TM domain from the membrane (Fig. 3A). In contrast, in the AtaA-TM–TpgA-N complex system, the number of contacts between AtaA-TM and the membrane was largely maintained, with only a slight decrease, until the point at which AtaA-TM–TpgA-N contacts dissociated (Fig. 3B). Importantly, this physical constraint implies that extraction of the AtaA-TM β-barrel can occur only after disruption of the AtaA-TM–TpgA-N interface. The persistence of AtaA-TM–TpgA-N contacts up to point e (Fig. 2C), which corresponds to the final dissociation of the AtaA-TM–TpgA-N interface, therefore provides a direct mechanistic explanation for the increased extraction force observed in the complex system. Beyond point e, further extraction led to a sharp decrease in AtaA-TM–membrane contacts (Fig. 3B). Similarly, the number of contacts between AtaA-TM and TpgA-N was maintained up to point e during the AtaA-TM extraction process. Notably, during this process, the number of contacts between TpgA-N and the membrane increased as AtaA-TM extraction progressed, with a significant increase observed in the 5- to 10-nm extension range, where large extraction forces were applied (Fig. 3B). These results suggest that local membrane deformation occurs around TpgA-N during AtaA-TM extraction and that increased membrane surface association of TpgA-N, without insertion into the membrane core, may contribute to resistance against membrane extraction of the AtaA-TM domain. To further clarify the nature of the increased TpgA-N–membrane contacts observed in Fig. 3B, we decomposed membrane-contact counts by component (POPE/POPG, lipid A, core oligosaccharide, and O-antigen) (Fig. S7). The increase in TpgA-N–membrane contacts during extraction was dominated by interactions with POPE/POPG, with a minor contribution from lipid A, whereas no detectable contacts with the core oligosaccharide or O-antigen were observed. These results indicate that TpgA-N primarily associates with headgroup region of inner leaflet lipids (POPE/POPG) rather than penetrating into the polysaccharide-rich LPS region.

**Fig. 4.**
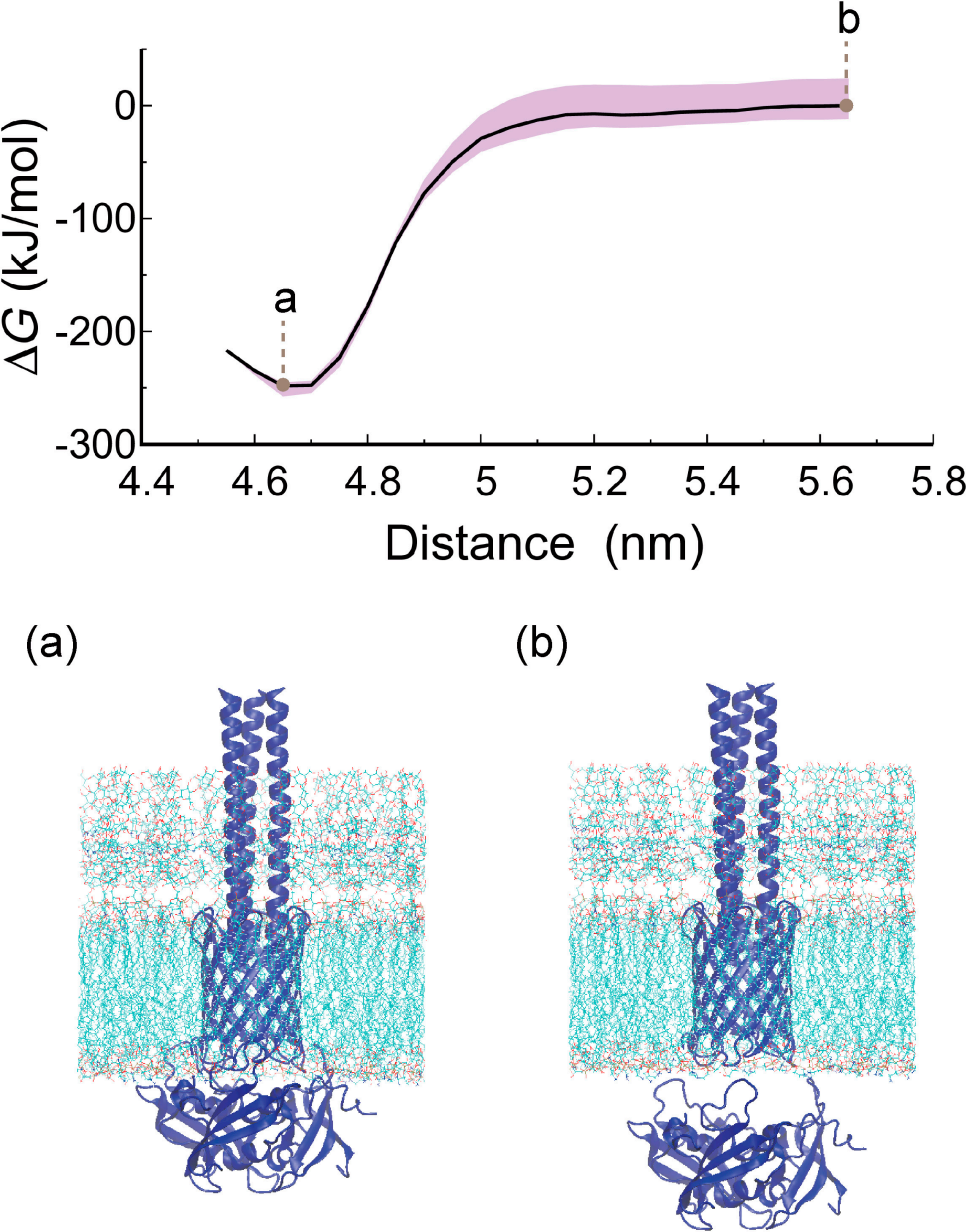
Restrained separation free energy profile of the AtaA-TM–TpgA-N complex. The free energy profile was calculated by thermodynamic integration of the window-averaged mean forces, using the COM separation between the AtaA-TM domain and TpgA-N along the membrane normal as the reaction coordinate. Mean forces were averaged over 2 to 30 ns for each of the 23 sampling windows. The black solid line represents the cumulative free energy profile, and the purple shaded region indicates the 95% confidence interval estimated from block-wise integration (4-ns blocks) using the 2.5th and 97.5th percentiles. Representative snapshots corresponding to (a) the free energy minimum and (b) the largest COM separation are shown. Water molecules and ions are omitted for clarity.

We next evaluated the stability of the AtaA-TM–TpgA-N interaction by thermodynamic integration along the COM separation coordinate under positional and orientational restraints. The calculated restrained separation free energy was −247.8 kJ mol^−1^ (Fig. 4; see Fig. S8 for convergence analysis). Because positional and orientational restraints were applied and no restraint-release corrections were included, this value does not represent a standard-state binding free energy in solution. Rather, it corresponds to the free energy change associated with separation along the defined reaction coordinate under the applied restraint scheme. Although this value is not directly comparable to experimental binding affinities, it supports substantial thermodynamic stabilization of the AtaA-TM–TpgA-N complex under the membrane-embedded conditions.

**Fig. 5.**
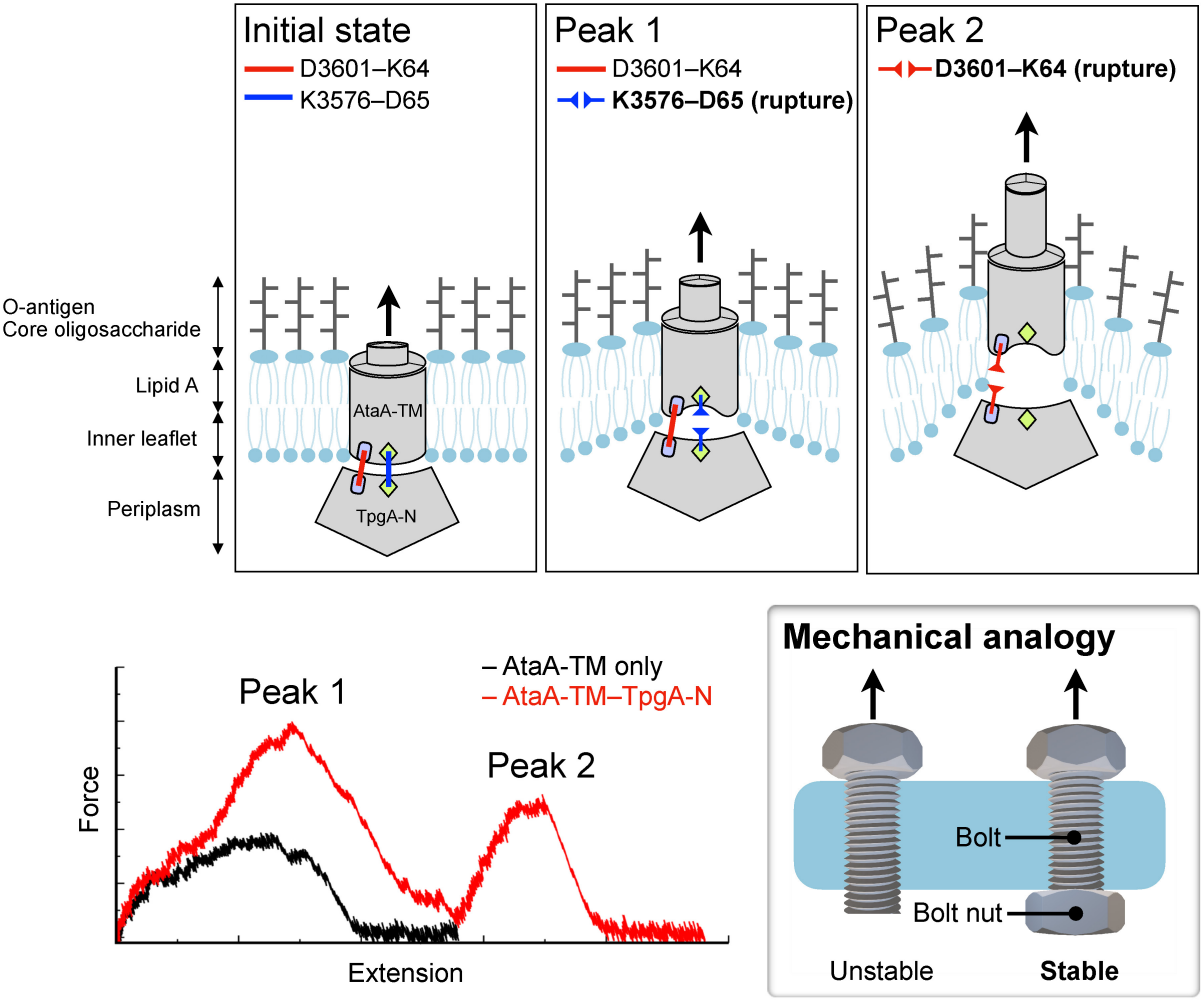
Schematic summary of the reinforcement mechanism of AtaA-TM anchoring by TpgA-N. Top panels illustrate the structural transitions during force-induced extraction of the AtaA-TM–TpgA-N complex from the outer membrane. In the initial state, 2 interfacial salt bridges (K3576–D65 and D3601–K64) stabilize the complex at the membrane–periplasm interface. Rupture of the K3576–D65 interaction corresponds to the first force peak, whereas subsequent rupture of the D3601–K64 interaction generates the second peak. The schematic also shows deformation of the surrounding membrane during extraction. Bottom left: Representative force–extension profiles from SMD simulations for AtaA-TM alone (black) and the AtaA-TM–TpgA-N complex (red), highlighting the appearance of 2 characteristic force peaks in the presence of TpgA-N. Bottom right: Conceptual mechanical analogy illustrating the stabilizing effect of TpgA-N. In this analogy, the AtaA transmembrane domain corresponds to a bolt passing through a plate, while the periplasmic protein TpgA acts analogously to a bolt nut that prevents the bolt from being pulled through the plate, thereby increasing resistance to force-induced extraction.

Previously, we reported that the AtaA-TM domain forms a stable complex with the periplasmic auxiliary protein TpgA, which is anchored to the peptidoglycan layer via a peptidoglycan-binding domain [21,40]. This architecture initially suggested that TpgA reinforces the membrane anchor by mechanically coupling AtaA-TM to the peptidoglycan layer. However, the enhanced extraction resistance observed in the present simulations indicates that periplasmic complex formation itself can provide substantial reinforcement even without peptidoglycan coupling. In well-studied TAAs, such as YadA and Hia [13,41,42], membrane anchoring has primarily been described based on intrinsic β-barrel translocator domains, and analogous TM–periplasmic partner interactions have not been reported. By contrast, bioinformatic analyses suggest that TAA–TpgA-like protein pairs are present across a broad range of genera, such as *Altererythrobacter*, *Burkholderia*, *Neisseria*, *Acinetobacter*, *Stenotrophomonas*, *Escherichia*, and *Campylobacter* [21]. Recently, a large-scale comparative analysis of TAAs further revealed that auxiliary proteins and C-terminal periplasmic extensions are widely distributed across bacterial lineages [43], suggesting that bacteria employ multiple strategies to support TAA assembly and anchoring stability, and that the AtaA–TpgA system represents one such strategy. This study provides the first direct mechanical and thermodynamic evidence that this type of complex can act as a potent reinforcement module for a TAA membrane anchor, highlighting the functional importance of periplasmic partner proteins in enabling giant bacterial adhesins to withstand extreme tensile loads. A schematic summary of the proposed reinforcement mechanism is presented in Fig. 5. In the AtaA–TpgA complex, tensile force applied to the extracellular stalk is transmitted to the membrane-embedded AtaA-TM domain, while the interaction with the periplasmic protein TpgA stabilizes the anchoring region. During force-induced extraction, the membrane undergoes deformation, while TpgA maintains contact with the TM domain, thereby redistributing mechanical stress across the membrane–protein interface. Sequential rupture of the interfacial salt bridges (K3576–D65 and D3601–K64) corresponds to the 2 characteristic force peaks observed in the simulations. This arrangement increases resistance to membrane extraction, analogous to the stabilizing effect of a bolt nut preventing pull-through of a bolt from a plate.

To place the simulated forces in a physiological context, we estimated the hydrodynamic drag acting on a bacterial cell under environmental flow conditions. In natural aquatic environments such as rivers, flow velocities can reach ~0.1 to 1 m s^−1^ [44]. Using Stokes’ law (F=6πμrv), with representative parameters for water viscosity (~10^−3^ Pa s) and bacterial size (effective radius ~0.5 μm), the hydrodynamic drag acting on a single bacterial cell is estimated to be on the order of ~1 to 10 nN. Although such estimates apply at the whole-cell level, they indicate that tensile loads in the nanonewton range are not implausible under strong hydrodynamic stress. During detachment events, the applied force may become concentrated on a limited number of adhesin molecules, potentially generating substantial tensile loads acting on individual adhesins. In this context, the peak forces observed in the SMD simulations (~3 to 4 nN) are within a physically plausible range. However, the present simulation setup is not intended to quantitatively reproduce physiological loading conditions. Instead, the simulations provide a comparative framework under identical pulling protocols that allows us to evaluate how TpgA binding increases the resistance of AtaA-TM to membrane extraction under tensile load.

This comparison also raises the question of which biological features of AtaA might make such reinforcement particularly important. One likely factor is the exceptional adhesiveness of AtaA itself. AtaA has been reported to exhibit exceptionally strong adhesion compared with canonical model TAAs such as YadA [45], suggesting that enhanced mechanical reinforcement may be required to sustain the higher tensile loads associated with such strong adhesion. This property is likely advantageous for environmental *Acinetobacter* sp. Tol 5, which inhabits dynamic aqueous environments where stable surface attachment under flow is essential. Membrane composition may also contribute to the reinforcement. The lipid and LPS composition of the outer membrane is known to vary among bacterial lineages [46], and such variation may influence membrane mechanical properties and anchoring stability, although this possibility was not examined in the present study. These predictions could be experimentally tested in future studies, for example, by mutational analysis of key interfacial residues, single-molecule force measurements using AFM, spectroscopic approaches such as electron paramagnetic resonance (EPR) to probe interfacial conformational changes, and by assessing whether anchoring stability depends on outer membrane composition using bacterial hosts (or engineered strains) with altered lipid and LPS profiles.

## Conclusions

In this study, we investigated the mechanical and thermodynamic stability of the AtaA-TM anchor in complex with the periplasmic auxiliary protein TpgA-N. All-atom SMD simulations showed that association with TpgA-N is accompanied by a marked increase in the resistance of the AtaA-TM domain to membrane extraction under tensile load. Compared with AtaA-TM alone, the AtaA-TM–TpgA-N complex exhibited higher peak extraction forces and distinct force–extension profiles, suggesting that complex formation alters the mechanical response of the TM anchor during extraction.

Because TpgA-N is a soluble, hydrophilic periplasmic protein, its translocation through the hydrophobic membrane core is physically prohibited. Consequently, membrane extraction of AtaA-TM is contingent upon the prior dissociation of TpgA-N. This physical constraint provides a mechanistic explanation for the enhanced extraction forces observed in the presence of TpgA-N.

Interfacial contact analyses further demonstrated that TpgA-N allows the interactions between AtaA-TM and the membrane to be maintained over a longer extraction distance. Additionally, the emergence of new contacts between TpgA-N and the membrane during extraction suggests that TpgA-N stabilizes the anchor by effectively modulating the distribution of interfacial stress. These mechanical observations are consistent with our thermodynamic integration results, which yielded a favorable restrained separation free energy for the AtaA-TM–TpgA-N complex, supporting stabilization of the interaction.

Together, our findings demonstrate that the formation of a periplasmic partner complex serves as an effective reinforcement for TAA membrane anchoring. More broadly, this work suggests that periplasmic auxiliary proteins may play a crucial role in enhancing the robustness of outer membrane anchoring, thereby enabling bacterial adhesins, particularly larger and highly adhesive ones, to withstand the substantial tensile forces encountered during surface attachment and environmental stress.

## Supplementary Materials

Supplementary 1 Figs. S1 to S8 Tables S1 to S4 Texts S1 to S7 Movies S1 to S5
