# Supplementary material for: Computational Analysis of Periplasmic Protein-Mediated Resistance to Membrane Extraction of a Trimeric Autotransporter Adhesin Transmembrane Domain
Source: Comput Struct Biotechnol J. 2026 Apr 8;35(1):0045. doi: 10.34133/csbj.0045 (PMC13082670; doi:10.34133/csbj.0045)
Supplement: Supplementary 1 — Figs. S1 to S8 Tables S1 to S4 Texts S1 to S7 Movies S1 to S5 [file csbj.0045.f1.zip › Revise_SI_TM-SMD.docx]

**Supporting Information**

**Computational analysis of periplasmic protein-mediated resistance to membrane extraction of a trimeric autotransporter adhesin transmembrane domain**

Jun Sasahara ^a^, Shogo Yoshimoto ^a^, Atsuo Suzuki ^a^, Katsutoshi Hori *^,a^

^a^Department of Biomolecular Engineering, Graduate School of Engineering, Nagoya University, Nagoya, Aichi 464-8603, Japan.

* Correspondence: Katsutoshi Hori

Tel: +81-52-789-3339

E-mail: khori@chembio.nagoya-u.ac.jp


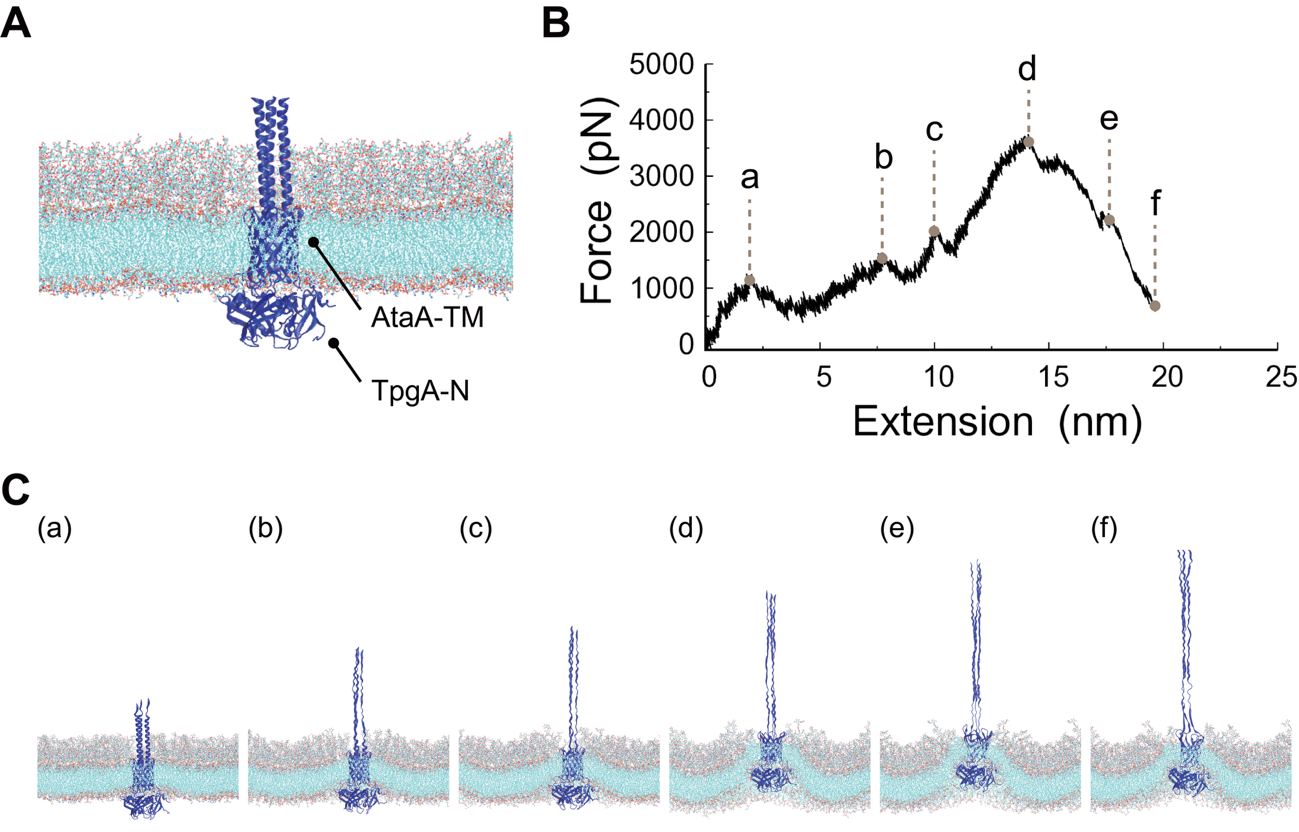


**Fig. S1.** SMD simulation of the full-length AtaA-TM model during membrane extraction. (A) Initial configuration of the full-length AtaA-TM domain embedded in a lipid bilayer. Water molecules and ions are omitted for clarity. (B) Force-extension profile obtained from SMD simulation in which the full-length AtaA-TM model was extracted along the membrane normal (Z-axis). (C) Structural snapshots corresponding to representative time points on the force-extension profile shown in (B). Prior to complete detachment of the AtaA-TM domain from the membrane, unfolding of the N-terminal coiled-coil region was observed. The pulling velocity was 2 nm ns^-1^.

**
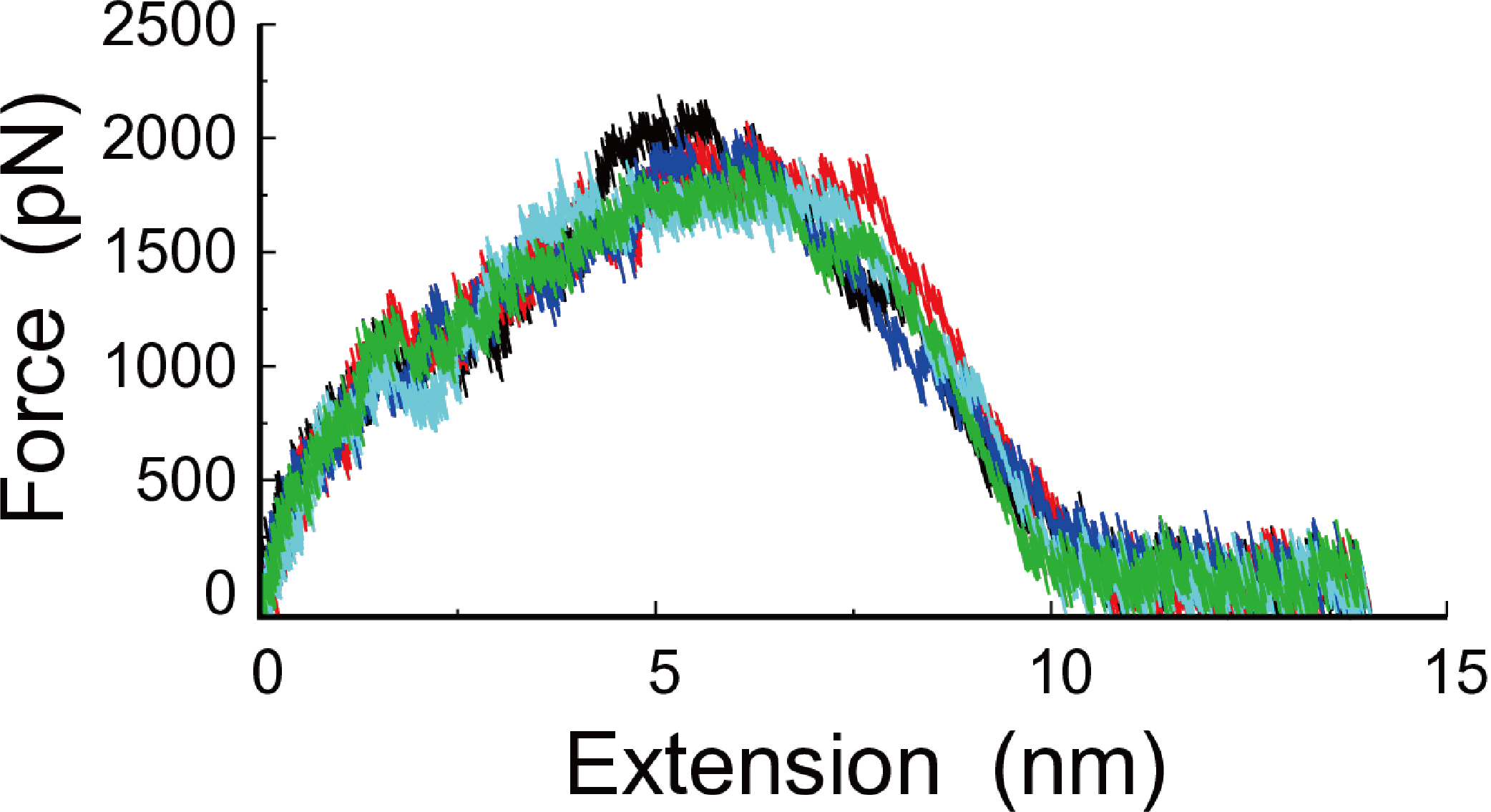
**

**Fig. S2.** Reproducibility of SMD simulations for the short-stalk AtaA-TM system. Force-extension profiles obtained from five independent SMD simulations (n = 5) of the short-stalk AtaA-TM system are shown in different colors. The pulling velocity was 2 nm ns^-1^.

**
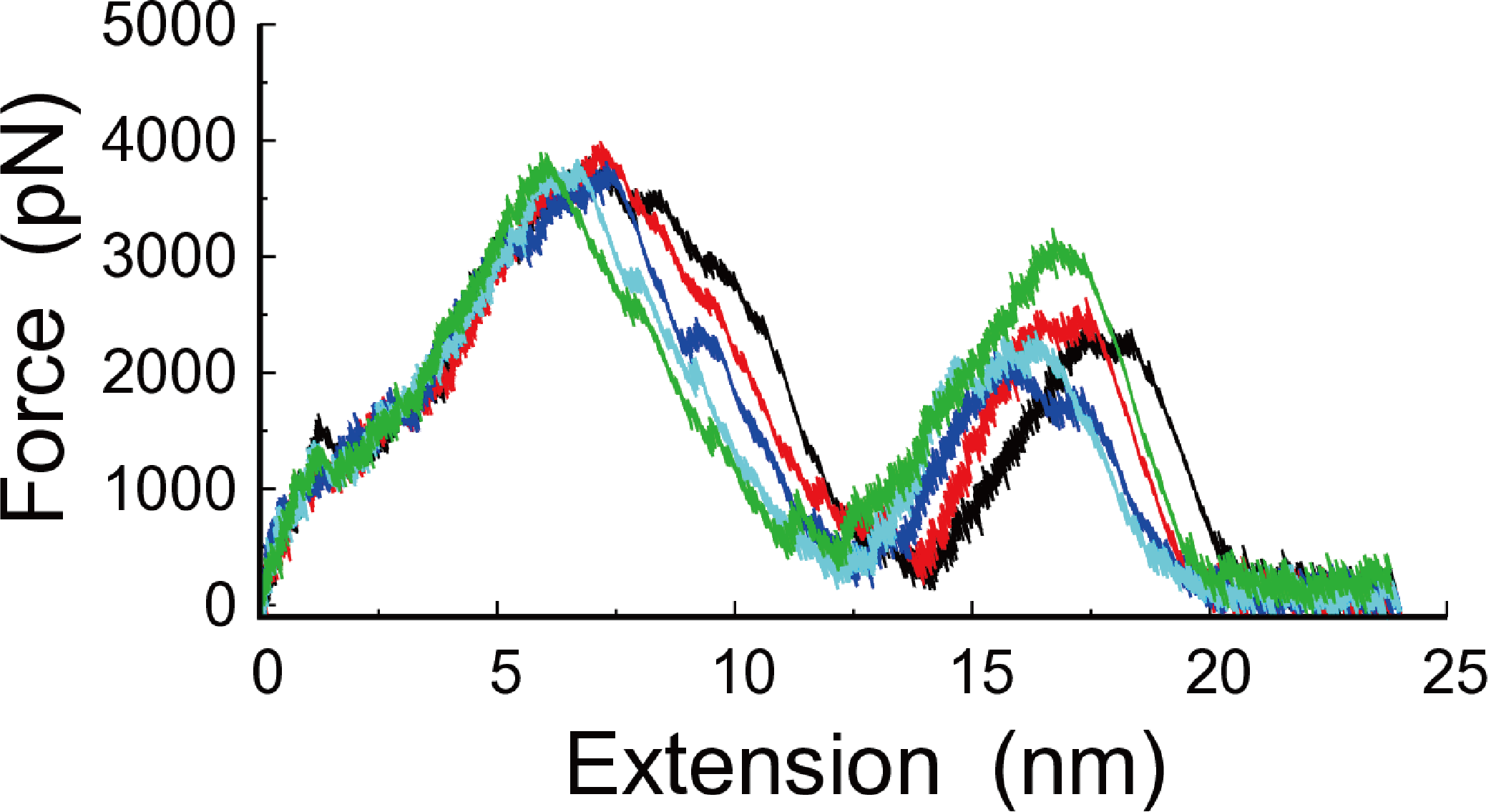
**

**Fig. S3.** Reproducibility of SMD simulations for the short-stalk AtaA-TM–TpgA-N complex. Force-extension profiles obtained from five independent SMD simulations (n = 5) of the short-stalk AtaA-TM–TpgA-N complex are shown in different colors. The pulling velocity was 2 nm ns^-1^.


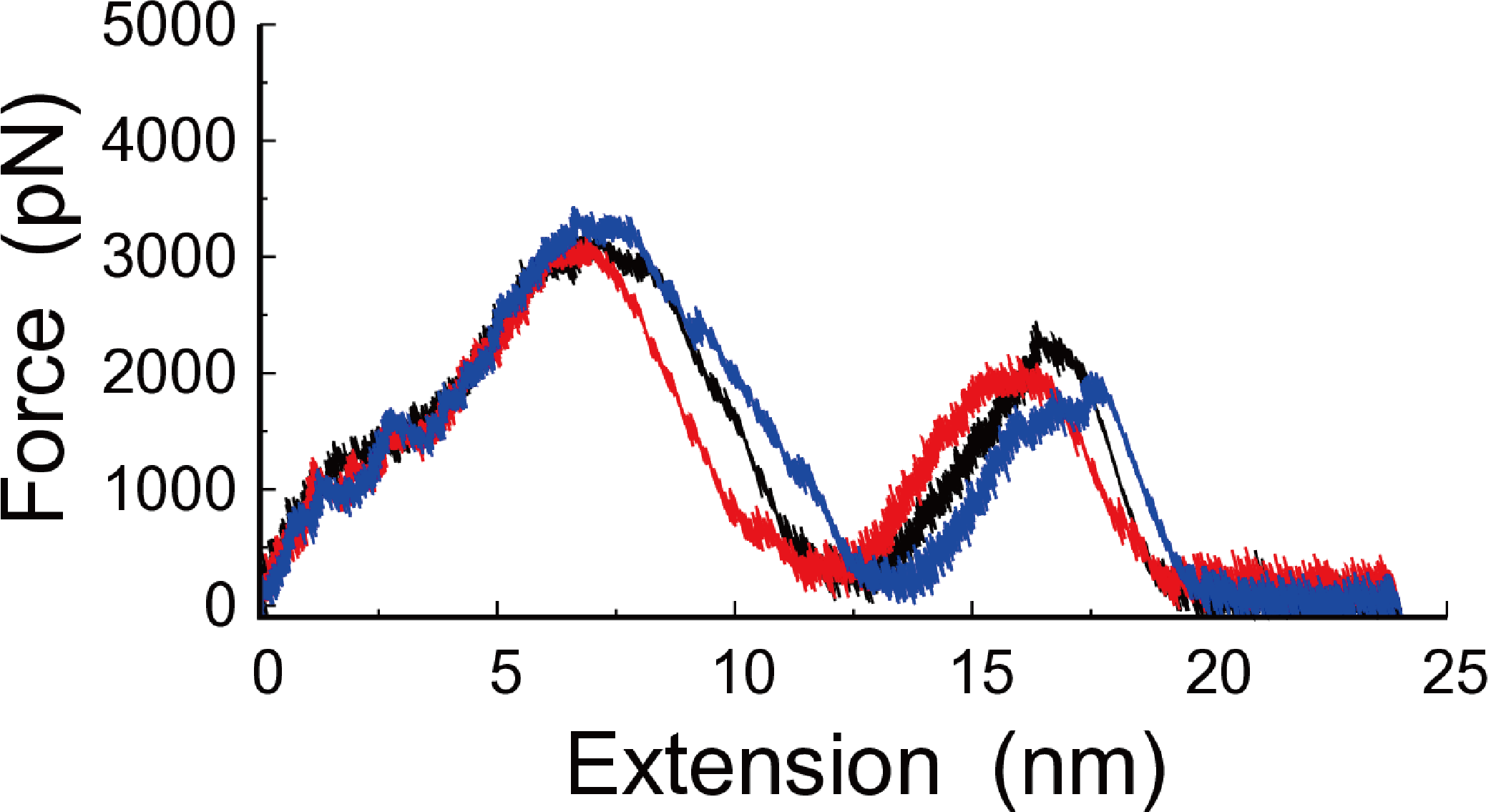


**Fig. S4.** Reproducibility of SMD simulations for the short-stalk AtaA-TM–TpgA-N complex. Force-extension profiles obtained from three independent SMD simulations (n = 3) of the short-stalk AtaA-TM–TpgA-N complex are shown in different colors. The pulling velocity was 1 nm ns^-1^. The maximum peak force was 3,270 ± 141 pN (mean ± SD, n = 3; 95% CI, 2,919–3,621 pN).

**
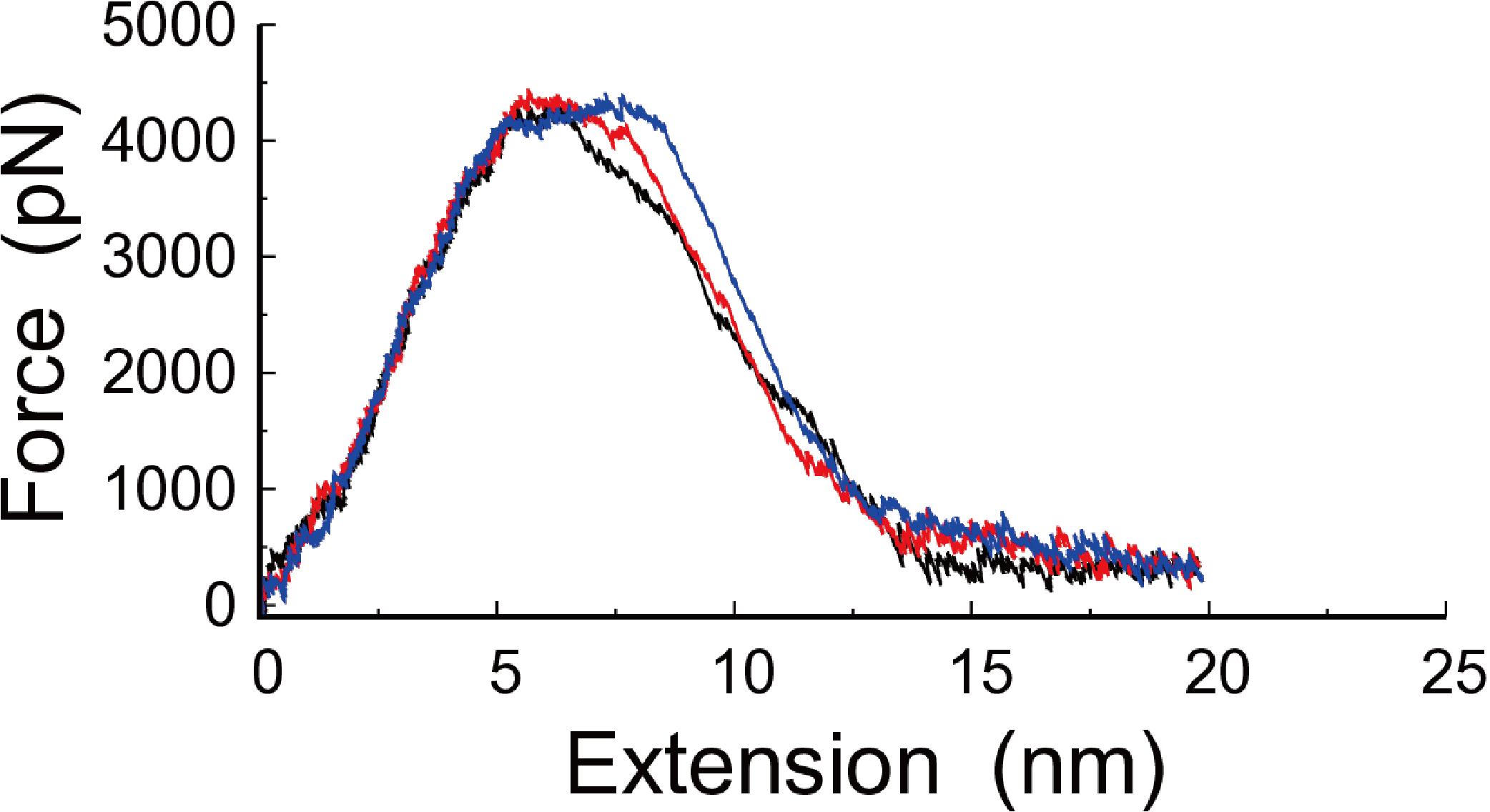
**

**Fig. S5.** Reproducibility of SMD simulations for the trimeric TpgA-N system. Force-extension profiles obtained from three independent SMD simulations (n = 3) of the trimeric TpgA-N system are shown in different colors. The pulling velocity was 2 nm ns^-1^.


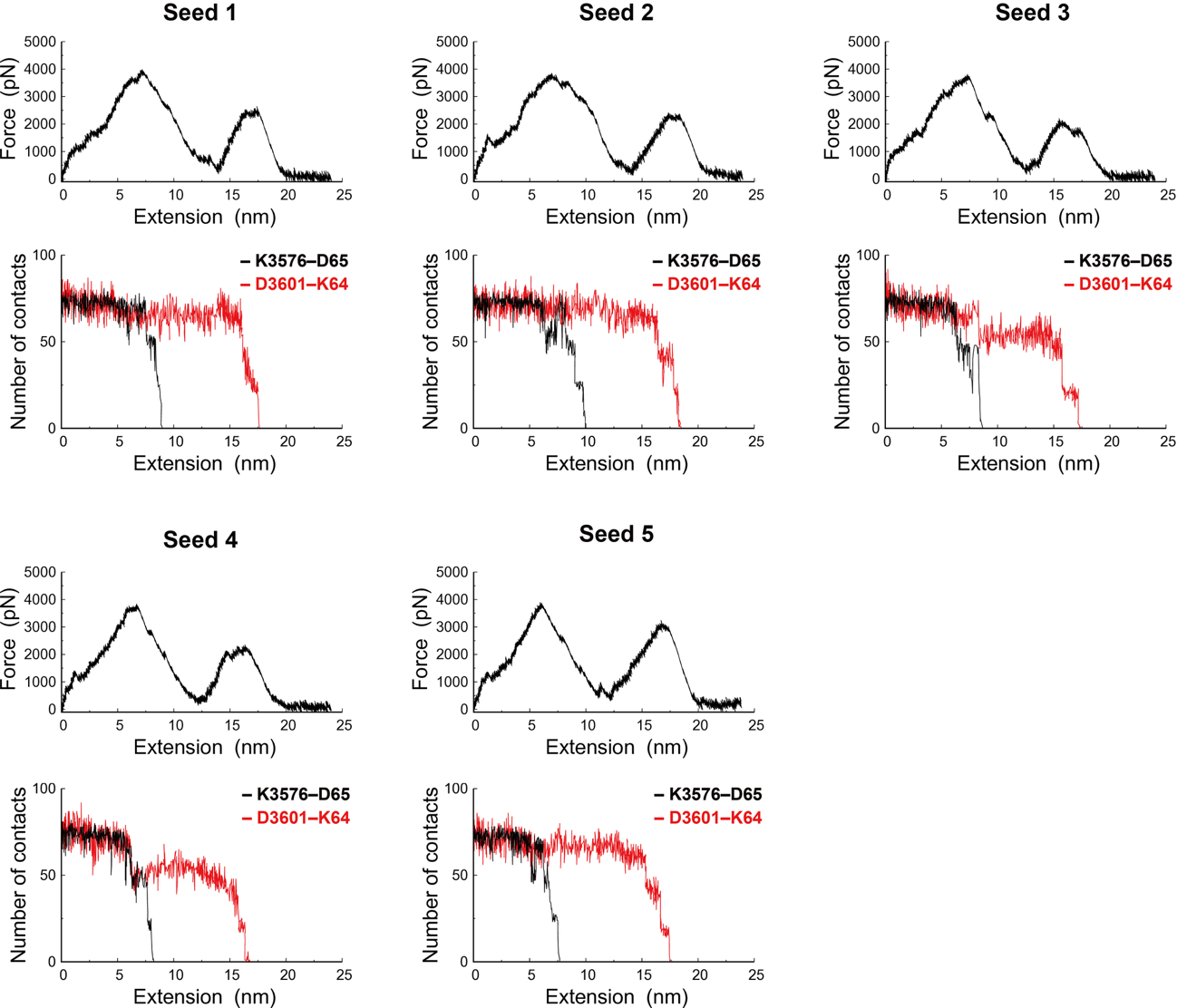


**Fig. S6.** Correlation between force peaks and salt-bridge rupture across independent trajectories. Force-extension curves (top) and corresponding contact numbers (bottom) for the K3576–D65 (black) and D3601–K64 (red) pairs are shown for five independent simulations (Seeds 1–5). In all trajectories, the K3576–D65 interaction ruptures at extensions corresponding to the first force peak, while the D3601–K64 interaction ruptures at extensions corresponding to the second peak, indicating a consistent sequential rupture mechanism. Contacts were defined as atomic pairs within 0.45 nm using the same criteria as in the main contact analysis.


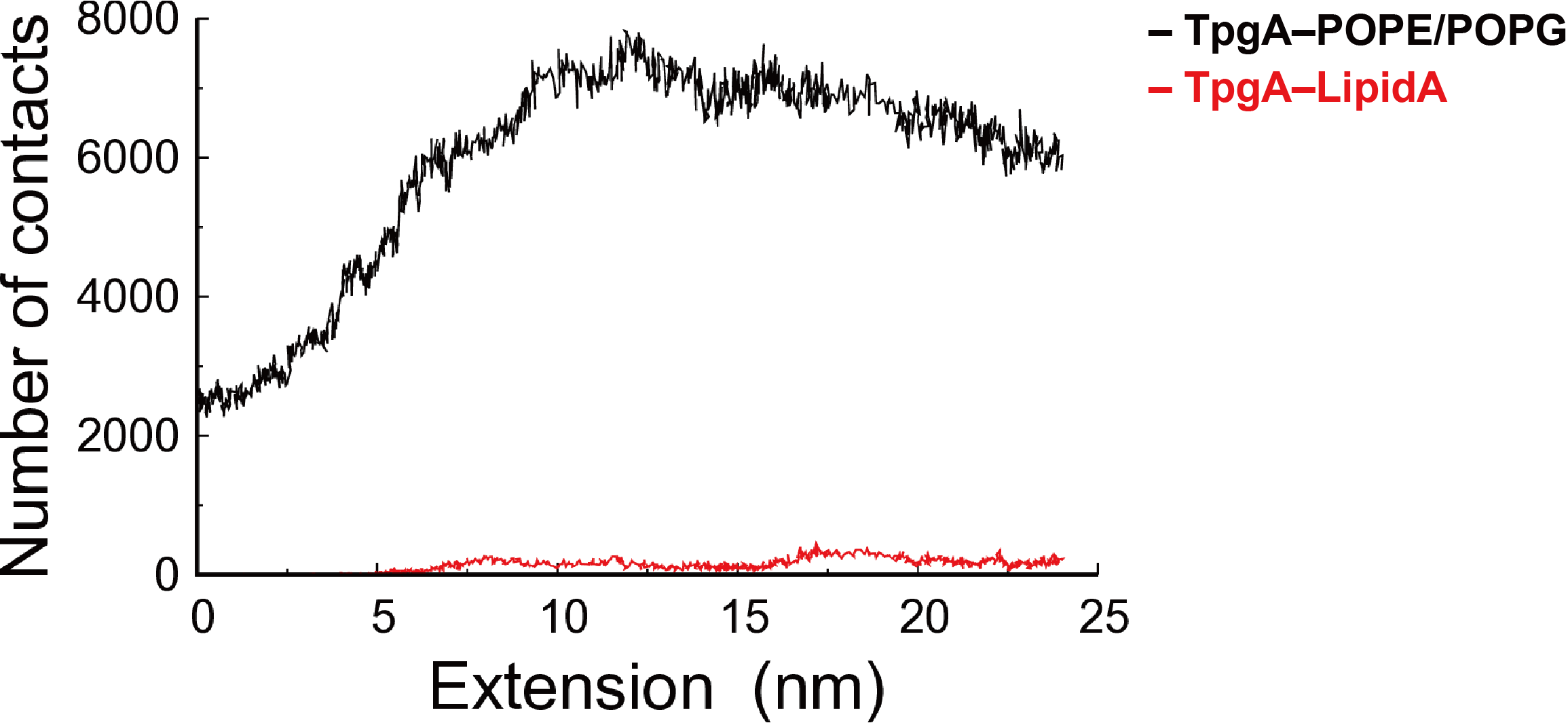


**Fig. S7.** Component-resolved analysis of TpgA-N–membrane contacts during extraction. Contacts between TpgA-N and POPE/POPG (black line) and lipid A (red line) are plotted against extension distance. Contacts between TpgA-N and the core oligosaccharide or O-antigen were not detected and are omitted.


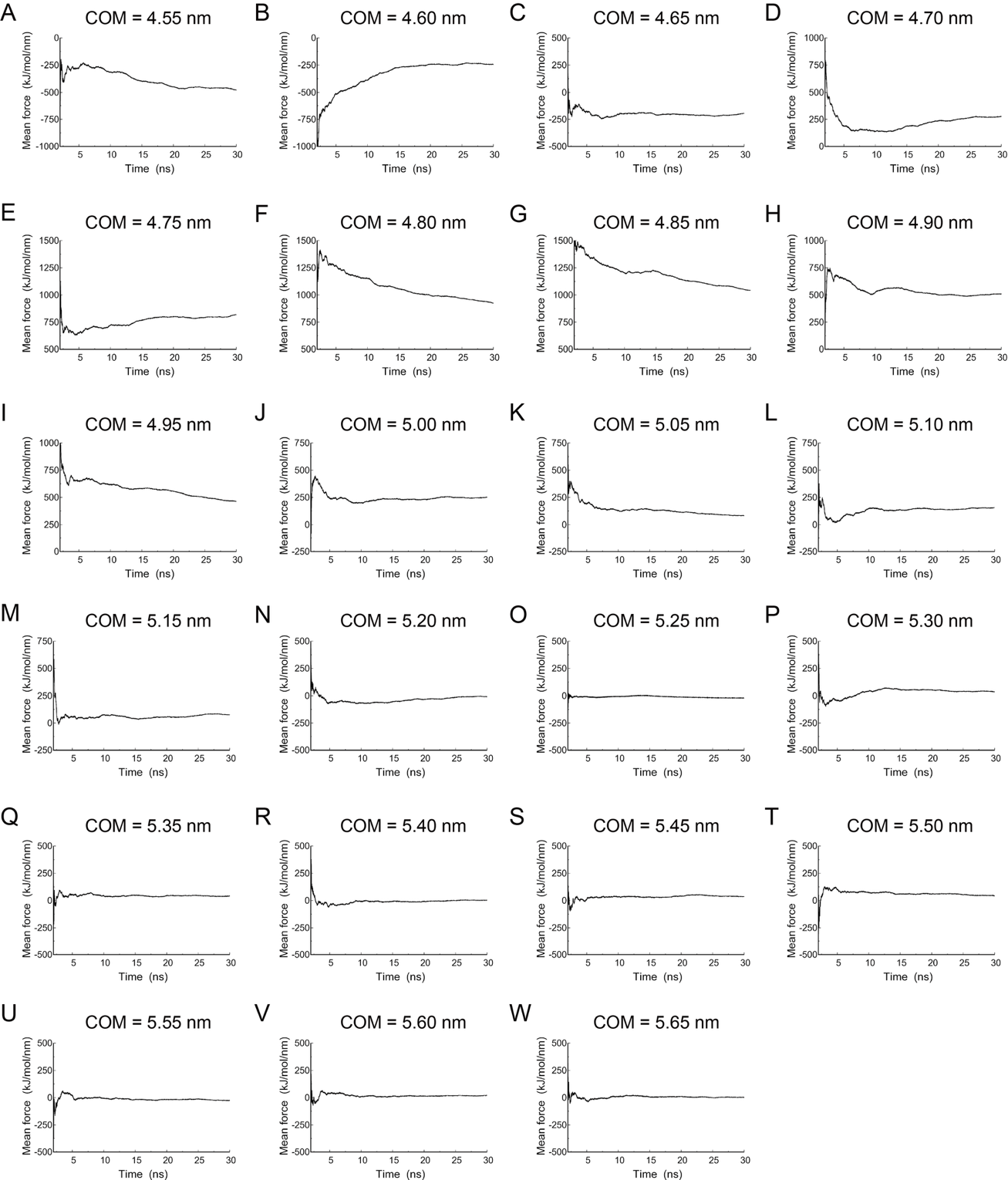


**Fig. S8.** Convergence of the cumulative average mean force in each window. Panels A–W correspond to individual windows with different center-of-mass (COM) distances between AtaA-TM and TpgA-N, as indicated in each panel (4.55–5.65 nm). For each window, the cumulative average of the mean force was calculated over 2–30 ns and is plotted against simulation time.

**Table S1.** Box size and number of atoms used in the MD simulations

| System | Box size (nm^3^) | Total atoms | Protein atoms | Water molecules |
| --- | --- | --- | --- | --- |
| AtaA-TM–TpgA-N | 15.0 × 15.0 × 80.0 | 1,752,593 | 9,312 | 544,348 |
| Short-stalk AtaA-TM | 15.1 × 15.1 × 80.0 | 1,768,467 | 3,024 | 550,917 |
| Short-stalk AtaA-TM–TpgA-N | 15.0 × 15.0 × 80.0 | 1,753,126 | 7,686 | 545,067 |
| TpgA-N | 15.0 × 15.0 × 80.0 | 1,755,077 | 4,662 | 544,548 |

**Table S2.** Box size and number of atoms used in the free energy calculation

| System | Box size (nm^3^) | Total atoms | Protein atoms | Water molecules |
| --- | --- | --- | --- | --- |
| AtaA-TM–TpgA-N | 8.1 × 8.1 × 16.5 | 107,893 | 9,312 | 23,914 |

**Table S3.** Equilibration process for simulation systems

| Step | Ensemble | Time (ps) | Description |
| --- | --- | --- | --- |
| 1 | – | – | Energy minimization |
| 2 | NVT | 125 | Equilibration stage 1 |
| 3 | NVT | 125 | Equilibration stage 2 |
| 4 | NPT | 125 | Equilibration stage 3 |
| 5 | NPT | 500 | Equilibration stage 4 |
| 6 | NPT | 500 | Equilibration stage 5 |
| 7 | NPT | 500 | Equilibration stage 6 |

**Table S4.** Random seeds for independent SMD simulations

| System | Replica | gen_seed |
| --- | --- | --- |
| Short-stalk AtaA-TM (2 nm ns^-1^) | 1 | 482091 |
|  | 2 | 159384 |
|  | 3 | 927405 |
|  | 4 | 310572 |
|  | 5 | 648219 |
| Short-stalk AtaA-TM–TpgA-N (2 nm ns^-1^) | 1 | 739102 |
|  | 2 | 516394 |
|  | 3 | 850213 |
|  | 4 | 127849 |
|  | 5 | 408736 |
| Short-stalk AtaA-TM–TpgA-N (1 nm ns^-1^) | 1 | 672938 |
|  | 2 | 134672 |
|  | 3 | 407836 |
| TpgA-N (2 nm ns^-1^) | 1 | 528174 |
|  | 2 | 193045 |
|  | 3 | 804612 |

**Text S1. Step 1 energy minimization mdp file**

define = -DPOSRES -DPOSRES_FC_BB=4000.0 -DPOSRES_FC_SC=2000.0 -DPOSRES_FC_LIPID=1000.0 -DDIHRES -DDIHRES_FC=1000.0

integrator = steep

emtol = 1000.0

nsteps = 5000

nstlist = 10

cutoff-scheme = Verlet

rlist = 1.2

vdwtype = Cut-off

vdw-modifier = Force-switch

rvdw_switch = 1.0

rvdw = 1.2

coulombtype = PME

rcoulomb = 1.2

constraints = h-bonds

constraint_algorithm = LINCS

**Text S2. Step 2 equilibration mdp file**

define = -DPOSRES -DPOSRES_FC_BB=4000.0 -DPOSRES_FC_SC=2000.0 -DPOSRES_FC_LIPID=1000.0 -DDIHRES -DDIHRES_FC=1000.0

integrator = md

dt = 0.001

nsteps = 125000

cutoff-scheme = Verlet

nstlist = 20

rlist = 1.2

vdwtype = Cut-off

vdw-modifier = Force-switch

rvdw_switch = 1.0

rvdw = 1.2

coulombtype = PME

rcoulomb = 1.2

tcoupl = v-rescale

tc_grps = System

tau_t = 1.0

ref_t = 301.15

constraints = h-bonds

constraint_algorithm = LINCS

nstcomm = 100

comm_mode = linear

comm_grps = System

gen-vel = yes

gen-temp = 301.15

gen-seed = -1

**Text S3. Step 3 equilibration mdp file**

define = -DPOSRES -DPOSRES_FC_BB=2000.0 -DPOSRES_FC_SC=1000.0 -DPOSRES_FC_LIPID=400.0 -DDIHRES -DDIHRES_FC=400.0

integrator = md

dt = 0.001

nsteps = 125000

cutoff-scheme = Verlet

nstlist = 20

rlist = 1.2

vdwtype = Cut-off

vdw-modifier = Force-switch

rvdw_switch = 1.0

rvdw = 1.2

coulombtype = PME

rcoulomb = 1.2

tcoupl = v-rescale

tc_grps = System

tau_t = 1.0

ref_t = 301.15

constraints = h-bonds

constraint_algorithm = LINCS

continuation = yes

nstcomm = 100

comm_mode = linear

comm_grps = System

**Text S4. Step 4 equilibration mdp file**

define = -DPOSRES -DPOSRES_FC_BB=1000.0 -DPOSRES_FC_SC=500.0 -DPOSRES_FC_LIPID=400.0 -DDIHRES -DDIHRES_FC=200.0

integrator = md

dt = 0.001

nsteps = 125000

cutoff-scheme = Verlet

nstlist = 20

rlist = 1.2

vdwtype = Cut-off

vdw-modifier = Force-switch

rvdw_switch = 1.0

rvdw = 1.2

coulombtype = PME

rcoulomb = 1.2

tcoupl = v-rescale

tc_grps = System

tau_t = 1.0

ref_t = 301.15

pcoupl = Parrinello-Rahman

pcoupltype = semiisotropic

tau_p = 5.0

compressibility = 4.5e-5 4.5e-5

ref_p = 1.0 1.0

refcoord_scaling = com

constraints = h-bonds

constraint_algorithm = LINCS

continuation = yes

nstcomm = 100

comm_mode = linear

comm_grps = System

**Text S5. Step 5 equilibration mdp file**

define = -DPOSRES -DPOSRES_FC_BB=500.0 -DPOSRES_FC_SC=200.0 -DPOSRES_FC_LIPID=200.0 -DDIHRES -DDIHRES_FC=200.0

integrator = md

dt = 0.002

nsteps = 250000

cutoff-scheme = Verlet

nstlist = 20

rlist = 1.2

vdwtype = Cut-off

vdw-modifier = Force-switch

rvdw_switch = 1.0

rvdw = 1.2

coulombtype = PME

rcoulomb = 1.2

tcoupl = v-rescale

tc_grps = System

tau_t = 1.0

ref_t = 301.15

pcoupl = Parrinello-Rahman

pcoupltype = semiisotropic

tau_p = 5.0

compressibility = 4.5e-5 4.5e-5

ref_p = 1.0 1.0

refcoord_scaling = com

constraints = h-bonds

constraint_algorithm = LINCS

continuation = yes

nstcomm = 100

comm_mode = linear

comm_grps = System

**Text S6. Step 6 equilibration mdp file**

define = -DPOSRES -DPOSRES_FC_BB=200.0 -DPOSRES_FC_SC=50.0 -DPOSRES_FC_LIPID=40.0 -DDIHRES -DDIHRES_FC=100.0

integrator = md

dt = 0.002

nsteps = 250000

cutoff-scheme = Verlet

nstlist = 20

rlist = 1.2

vdwtype = Cut-off

vdw-modifier = Force-switch

rvdw_switch = 1.0

rvdw = 1.2

coulombtype = PME

rcoulomb = 1.2

tcoupl = v-rescale

tc_grps = System

tau_t = 1.0

ref_t = 301.15

pcoupl = Parrinello-Rahman

pcoupltype = semiisotropic

tau_p = 5.0

compressibility = 4.5e-5 4.5e-5

ref_p = 1.0 1.0

refcoord_scaling = com

constraints = h-bonds

constraint_algorithm = LINCS

continuation = yes

nstcomm = 100

comm_mode = linear

comm_grps = System

**Text S7. Step 7 equilibration mdp file**

define = -DPOSRES -DPOSRES_FC_BB=50.0 -DPOSRES_FC_SC=0.0 -DPOSRES_FC_LIPID=0.0 -DDIHRES -DDIHRES_FC=0.0

integrator = md

dt = 0.002

nsteps = 250000

cutoff-scheme = Verlet

nstlist = 20

rlist = 1.2

vdwtype = Cut-off

vdw-modifier = Force-switch

rvdw_switch = 1.0

rvdw = 1.2

coulombtype = PME

rcoulomb = 1.2

tcoupl = v-rescale

tc_grps = System

tau_t = 1.0

ref_t = 301.15

pcoupl = Parrinello-Rahman

pcoupltype = semiisotropic

tau_p = 5.0

compressibility = 4.5e-5 4.5e-5

ref_p = 1.0 1.0

refcoord_scaling = com

constraints = h-bonds

constraint_algorithm = LINCS

continuation = yes

nstcomm = 100

comm_mode = linear

comm_grps = System

**Movies**

**Movie S1.** Visualization of the extraction process in the SMD simulation of the full-length AtaA-TM–TpgA-N complex. The complex was embedded in a lipid bilayer and extracted along the membrane normal (Z-axis). Prior to complete detachment from the membrane, pronounced unfolding of the N-terminal coiled-coil region of AtaA was observed. The pulling velocity was 2 nm ns^-1^.

**Movie S2.** SMD simulation of the short-stalk AtaA-TM system. The short-stalk AtaA-TM model, lacking the N-terminal coiled-coil region, was embedded in a lipid bilayer and extracted along the membrane normal (Z-axis). During extraction, the β-barrel structure of the TM domain remained largely intact while lipid–protein interactions were gradually disrupted. This movie corresponds to the simulation shown in Fig. 1. The pulling velocity was 2 nm ns^-1^.

**Movie S3.** SMD simulation of the short-stalk AtaA-TM–TpgA-N complex. The complex was embedded in a lipid bilayer and extracted along the membrane normal (Z-axis). During extraction, AtaA-TM and TpgA-N moved cooperatively while maintaining their interfacial interactions, resulting in prolonged retention within the membrane compared with the short-stalk AtaA-TM system. This movie corresponds to the simulation shown in Fig. 2. The pulling velocity was 2 nm ns^-1^.

**Movie S4.** SMD simulation of the short-stalk AtaA-TM–TpgA-N complex performed at a reduced pulling velocity of 1 nm ns^-1^. The complex was embedded in a lipid bilayer and extracted along the membrane normal (Z-axis). Compared with simulations at higher pulling velocity, the peak force was reduced while the overall extraction behavior and force-extension profile remained qualitatively consistent (Fig. S4).

**Movie S5.** SMD simulation of the trimeric TpgA-N pulled through the membrane. The trimeric TpgA-N was embedded adjacent to a lipid bilayer and pulled along the membrane normal (Z-axis) to drive membrane penetration. During the simulation, substantial resistance to membrane entry was observed, and large forces were required to force TpgA-N into the bilayer. The pulling velocity was 2 nm ns^-1^.
